# Lumbar facet joint denervation targeting the medial branch in the sub-mammillary fossa: An anatomical optimization study

**DOI:** 10.1016/j.inpm.2025.100586

**Published:** 2025-04-22

**Authors:** John Tran, Aaron Conger, Keaton Lightfoot, Zachary L. McCormick, Eldon Loh

**Affiliations:** a Department of Physical Medicine and Rehabilitation, Western University, London, Canada; b Division of Anatomy, Department of Surgery, University of Toronto, Toronto, Canada; c Department of Physical Medicine and Rehabilitation, University of Utah School of Medicine, Salt Lake City, UT, USA; d Parkwood Institute Research, Lawson Research Institute, London, Canada

**Keywords:** Radiofrequency ablation, Neurotomy, Low back pain, Medial branch nerve

## Abstract

**Introduction:**

Recent anatomical studies have identified the sub-mammillary fossa as a potential target site to extend the length and more reliably capture the medial branch during lumbar facet joint denervation. Although a clinical case series was published describing positive outcomes targeting the sub-mammillary fossa, the ideal location for radiofrequency cannula placement has not been assessed. Further anatomical investigation of this novel technique is warranted to refine fluoroscopic landmarks for optimal placement.

**Methods:**

Twelve cannulae were placed under fluoroscopic guidance targeting the L3, L4, & L5 medial branches in 2 embalmed cadaveric specimens. Dissection, digitization, and high-fidelity 3D modelling methodology was used to identify fluoroscopic landmarks. Lesion simulation was performed on 3D models to analyze nerve capture.

**Results:**

In 5 of 12 placements (41.7 %), the medial branch capture rate was classified as “complete,” as the simulated lesion overlapped with the medial branch trunk or all of its distal branches. In 4 of 12 placements (33.3 %), the nerve capture rate was “partial” with at least one distal branch found beyond the boundary of the simulated lesion. In the remaining 3 placements (25.0 %), the capture rate was classified as “none,” as the medial branch trunk and all distal branches transited beyond the simulated lesion boundary. Refined fluoroscopic landmarks proposed were the lateral boundary of mammillary process (AP view); the mamillo-accessory notch/inferior boundary of facet joint line (oblique view); and the inferior aspect of the mammillary process (lateral view).

**Conclusions:**

This anatomy optimization study used dissection, imaging correlation, and high-fidelity modelling to assess cannula placement for capture of the medial branch at the sub-mammillary fossa for lumbar facet joint denervation. Based on the present analysis, refined fluoroscopic landmarks were proposed for further investigation.

## 1 Introduction

Detailed anatomical knowledge is essential to optimize image-guided pain procedures. Lumbar medial branch (MB) radiofrequency ablation (RFA) is a common procedure used to manage facetogenic low back pain. However, the quality of pain relief and duration of benefit following lumbar MB RFA may be variable due to challenges with patient selection and less than optimal cannula placement techniques [[1], [2], [3]]. To achieve more consistent outcomes, investigators have focused on optimizing the technical performance of lumbar MB RFA from an anatomical perspective [[4], [5], [6], [7], [8]]. It is conceivable that maximizing the length of the MB that is captured during RFA will contribute to more consistent and prolonged patient outcomes.

**Fig. 1:**
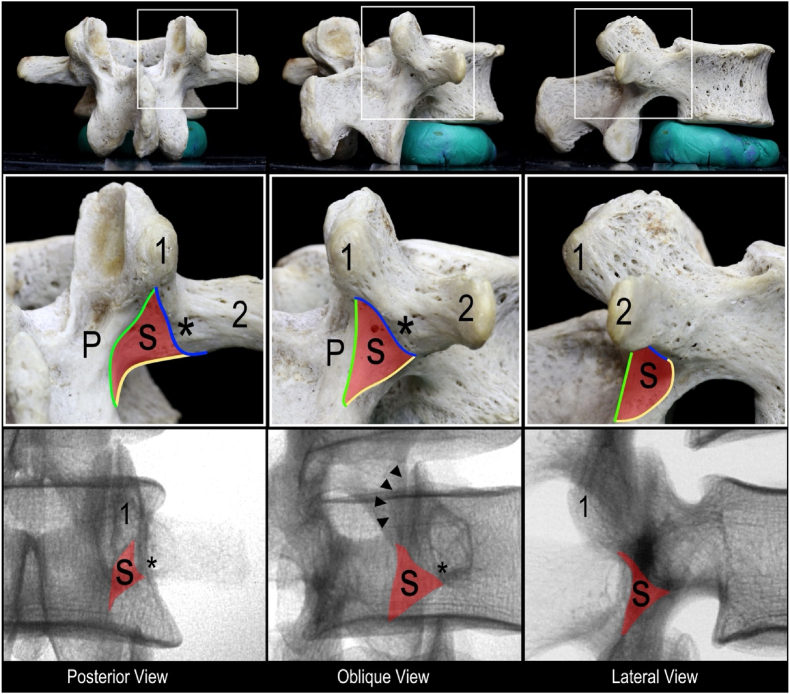
**Bony anatomy and fluoroscopic appearance of the sub-mammillary fossa.** Black arrowheads indicate contour of the mammillary process (1); 2, transverse process; asterisks, accessory process; S, sub-mammillary fossa (highlighted with red); P, region between the superior and inferior articular processes (pars interarticularis). Boundaries of the sub-mammillary fossa: pars interarticularis (medial, green curve), mamillo-accessory notch (superolateral, blue curve), junction between base of transverse process and inferior articular process (inferolateral, yellow curve).

Recent anatomical studies have investigated the relationship of the lumbar MB to surrounding bony and soft-tissue landmarks [7,[9], [10], [11]]. Emerging from this research was the identification of the sub-mammillary fossa as a novel supplementary target site to extend the length of the MB being captured (Fig. 1) [10]. The sub-mammillary fossa provides a distinct and complementary target to conventional approaches, which may be particularly valuable in cases of severe scoliosis or advanced facet hypertrophy. Expanding the available target options in cases where traditional landmarks are obscured or less accessible may improve the likelihood of successful denervation. Although a clinical case series was published describing positive outcomes following RFA at the sub-mammillary fossa and the lateral neck of the superior articular process (SAP), the ideal location for cannula placement at the sub-mammillary fossa has not been assessed [12]. Further anatomical investigation of the novel technique targeting the sub-mammillary fossa is warranted to identify the ideal fluoroscopic landmarks for optimal placement, maximizing the reliability of nerve capture and clinical outcomes. Therefore, the objectives of the current study were to: 1) perform fluoroscopy-guided cannula placements targeting the lumbar MB at the sub-mammillary fossa in a cadaveric model; 2) assess the accuracy of the fluoroscopic landmark to capture the lumbar MB using a dissection-based high-fidelity 3D modelling methodology; and 3) refine fluoroscopic landmarks to optimize cannula placement targeting the medial branch within the sub-mammillary fossa.

## 2 Materials and methods

Twelve radiofrequency cannulas (“cannulae”) were placed under fluoroscopic guidance targeting the L3, L4, & L5 MBs bilaterally in 2 lightly embalmed cadaveric specimens. Specimens with visible signs of previous surgery or severe spinal deformities were excluded. This study was approved by the University of Toronto Health Sciences Research Ethics Board (Protocol #27210).

### 2.1 Fluoroscopic-guided needle placement technique

The fluoroscopic-guided procedures were completed at the University Health Network Animal Resources Centre (ARC) located in the Princess Margaret Cancer Research Tower, Toronto, Ontario, Canada, with the assistance of a medical radiology technician. Cadaveric specimens were placed in a prone position and imaged using a fluoroscope. As there is no prior anatomical research to inform the best approach, cannula positioning and trajectory was deemed satisfactory as long as the tip was located within the sub-mammillary fossa. All 12 cannula placements, targeting the sub-mammillary fossa, used a coaxial approach either on an anteroposterior (AP) or oblique view. A detailed description of each technique is provided below. Following fluoroscopic-guided placement, each cannula was secured into the bone with a rubber mallet. This ensured the cannula was fixed and did not move during the subsequent dissection and digitization process. Fluoroscopic images were stored for subsequent correlational analysis with dissection data.

### 2.2 Coaxial cannula placement on AP view

Seven coaxial cannula placements were performed using an AP view. An AP view (with the superior vertebral endplate squared at the spinal segment of interest) was obtained to identify the sub-mammillary fossa located below the inferior margin of the mammillary process. Using a coaxial approach, a 17g cannula was then advanced toward the sub-mammillary fossa until bone contact. A 30-degree ipsilateral oblique view was obtained to confirm that the cannula tip was located medial and inferior to the mamillo-accessory notch (i.e., within the sub-mammillary fossa). A lateral view was then obtained to substantiate that the cannula was in contact with the periosteum, inferior to the mammillary process. This was performed at the L3, L4, and L5 MB levels.

### 2.3 Coaxial cannula placement on oblique view

In 5 cases, coaxial cannula placement was performed in the oblique view. After the superior vertebral endplate was squared at spinal segment of interest on AP view, a 30-degree ipsilateral oblique view was obtained to visualize the mamillo-accessory notch. The cannulae were then advanced toward the sub-mammillary fossa (located inferior and medial to the mamillo-accessory notch) on the oblique view until bone contact. AP and lateral views were obtained to confirm the cannulae tip were placed inferior to the mammillary process and in contact with the periosteum, respectively. In one specimen, the left mamillo-accessory notch of the L4 vertebral was not clearly visible, and hence, a coaxial placement in the AP view was used to target the sub-mammillary fossa.

### 2.4 Dissection, digitization, and high-fidelity 3D modeling

Following cannula placement, the specimens were transported to the University of Toronto Division of Anatomy for meticulous dissection. To expose the L3, L4, and L5 MBs and assess their spatial relationship to each cannula, skin and surrounding soft tissues were carefully removed. Next, the superficial back muscles were excised to expose the erector spinae muscle group. The erector spinae group was dissected sequentially while preserving the position of the cannulae. Muscle tissue was carefully dissected away to expose the lateral and intermediate branches of the lumbar dorsal ramus. The branches were traced proximally to the origin of the MB. The MB was then meticulously traced distally, and fiber bundles of the multifidus were excised to expose the course of the nerve deep to the muscle. Further removal of the multifidus exposed the branches of the MB and their relationship to the cannula tip and lumbar facet joints. Photographs were taken throughout the dissection process. Following dissection, the course of each MB, the cannulae, and bony landmarks (i.e. facet joint, mammillary process and transverse process) were digitized with a Microscribe G2X digitizer (Revware Inc, Raleigh, North Carolina). The digitized bony landmarks allowed for subsequent alignment of digitized data with a high-resolution scan of the bony surfaces and ligamentous structures of the lumbar spine and pelvic girdle. The high-resolution surface scan was generated using a Faro Laser ScanArm (FARO Technologies, Lake Mary, Florida) after the specimen was skeletonized by removing all soft tissues excluding ligaments and joint capsules stabilizing the lumbar spine and pelvic girdle. The digitized data of the nerves and cannulae were combined with the bony surface scan in Blender3D, an open-source 3D software, to generate high-fidelity 3D models. A spherical lesion with a diameter of 9.9 mm was simulated at the tip of the cannula to visualize the MB nerve capture rate when using the sub-mammillary fossa targeting technique. The simulated lesion was based on previously published data defining the geometry of an internally-cooled monopolar radiofrequency (RF) cannula [13]. The dimension of an internally-cooled monopolar RF lesion was used to maximize simulated nerve capture at the needle tip, due to the perpendicular placement. The expanded lesion geometry of internally-cooled monopolar RF lesion is ideal for perpendicular approaches as it ensures capture of the nerve target regardless of cannula trajectory (angle independence).

### 2.5 Data analysis

The extent of nerve capture within the sub-mammillary fossa was quantified using the high-fidelity 3D models and lesion simulation. Based on the overlap of the MB with the simulated lesion, nerve capture was categorized as “complete” (all branches within the lesion volume), “partial” (at least one branch), or “none” (no branches). The capture rate was reported as a percentage. Detailed analysis of dissected specimens, high-fidelity 3D models, nerve capture rate, and correlation with fluoroscopic imaging was performed. The positional relationship of the MB with the cannula and bony landmarks in the dissected specimens were used to identify fluoroscopic landmarks to refine cannula placement in order to optimize the reliability of capture of the entire MB within the sub-mammillary fossa.

## 3 Results

**Fig. 2:**
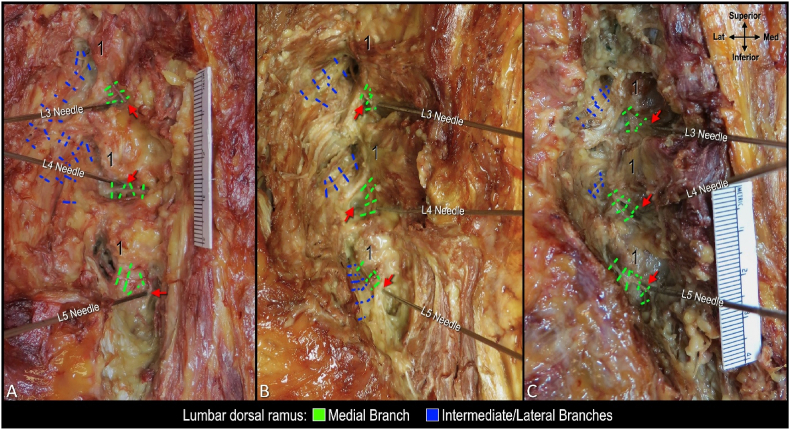
**Cadaveric dissections following cannula placement targeting third (L3), fourth (L4), and fifth (L5) lumbar medial branches. Oblique views.** A. Dissection showing relationship of medial branches to cannulae coaxially placed on a 30^o^ oblique view to target the sub-mammillary fossa. B-C. Dissection of medial branches and cannulae following coaxial placement in AP view. Note needle trajectories in B and C are different from A as these were placed coaxially in AP view. Red arrow indicates tip of cannula; 1, mamillary process.

**Fig. 3:**
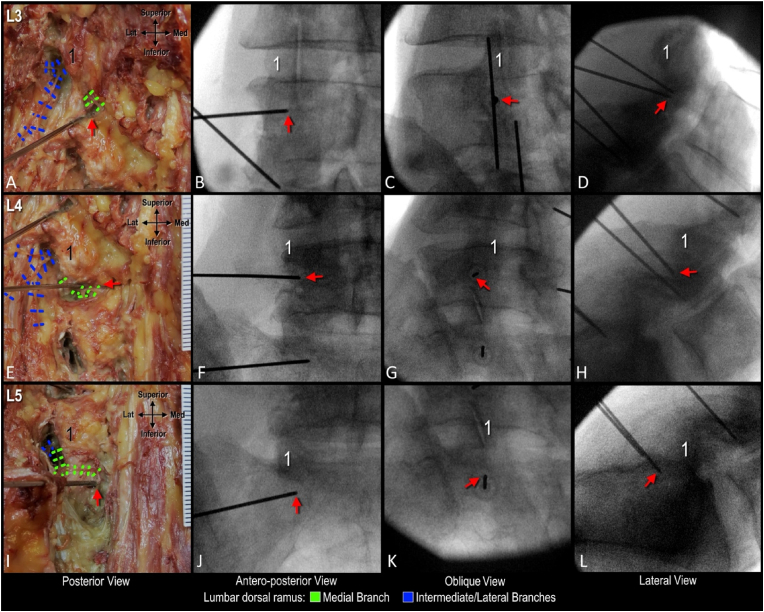
**Correlation of anatomy and fluoroscopy following coaxial cannula placement targeting sub-mammillary fossa on a 30° oblique view.** A. Dissection of cannula targeting L3 medial branch. B-D. Corresponding fluoroscopic image with superior endplate of L4 vertebra squared. E. Dissection of cannula targeting L4 medial branch. F-H. Corresponding fluoroscopic image with superior endplate of L5 vertebra squared. F. Dissection of cannula targeting L5 medial branch. J-L. Corresponding fluoroscopic image with body of sacrum squared. Red arrow indicates cannula tip; 1, mammillary process.

Twelve cannulae were successfully placed in the cadaveric specimens using fluoroscopic guidance. Each cannula was dissected, and its relationship to the corresponding lumbar MB was successfully exposed (Fig. 2). There was variability in the position of the distal end of the tip of each cannula relative to the MB and mammillary process (Fig. 3, red arrows).

**Fig. 4:**
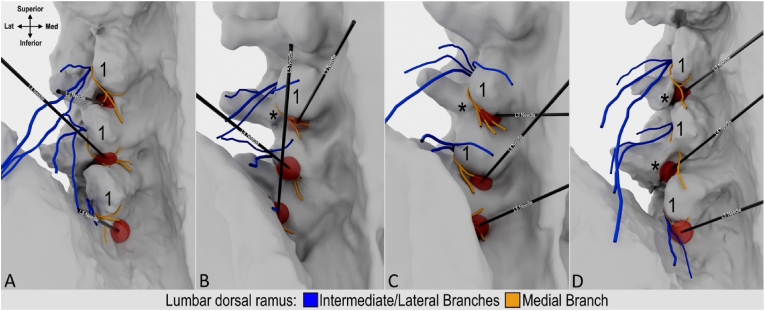
**High-fidelity 3D models with lesion simulation.** A and B. Relationship of medial branch to simulated lesions following coaxial cannula placement in oblique view. Note in panel B L3 needle was placed coaxially in AP view and L5 needle coaxially in less oblique trajectory. C and D. 3D models showing variability of medial branches and relationship to simulated lesion/cannula placed coaxially in AP view.1 indicates mammillary process; asterisk, accessory process.

**Fig. 5:**
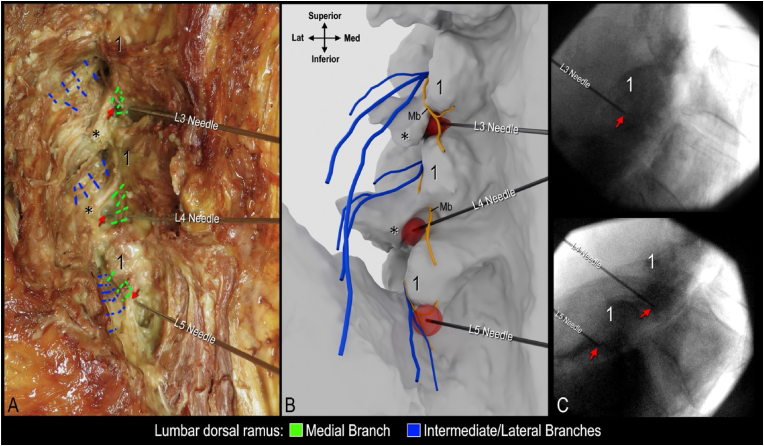
**Dissection, high-fidelity 3D modelling and fluoroscopy correlation following coaxial cannulae placement in AP view.** A. Dissection of L3, L4, and L5 medial branches (Mb). B. High-fidelity model with lesion simulation to visualize nerve capture. C. Lateral fluoroscopic view of cannula targeting L3 (top panel) and L4/L5 (bottom panel) medial branches. Red arrow indicates cannula tip; 1, mammillary process; asterisk, accessory process.

Three-dimensional visualization of the cannula tip and simulated lesion enabled the classification and quantification of nerve capture for all 12 placements (Fig. 4, Fig. 5). In 5 of 12 placements (41.7 %) the MB capture rate was classified as complete, as the simulated lesion overlapped with the trunk or all branches (Fig. 4A, L4 needle). In 4 of 12 placements (33.3 %) the MB capture rate was partial, as at least one branch was located beyond the boundary of the simulated lesion (Fig. 4C, L4 needle). In these cases, the cannula placements were found to be too far medial relative to the mammillary process (Fig. 2C, L3 needle). In the remaining 3 placements (25.0 %), the capture rate was classified as none, as the MB trunk and all branches were located beyond the boundary of the simulated lesion (Fig. 5B, L3 and L4 needles). The cannula placements in these cases were found to be too inferior to the mammillary process (Fig. 3, Fig. 5C, L4 needle). This resulted in the simulated lesion being placed anterior to the MB when the distal end of cannula tip was in contact with bone within the distal aspect of the sub-mammillary fossa (Fig. 4D, L3 and L4 needles).

**Fig. 6:**
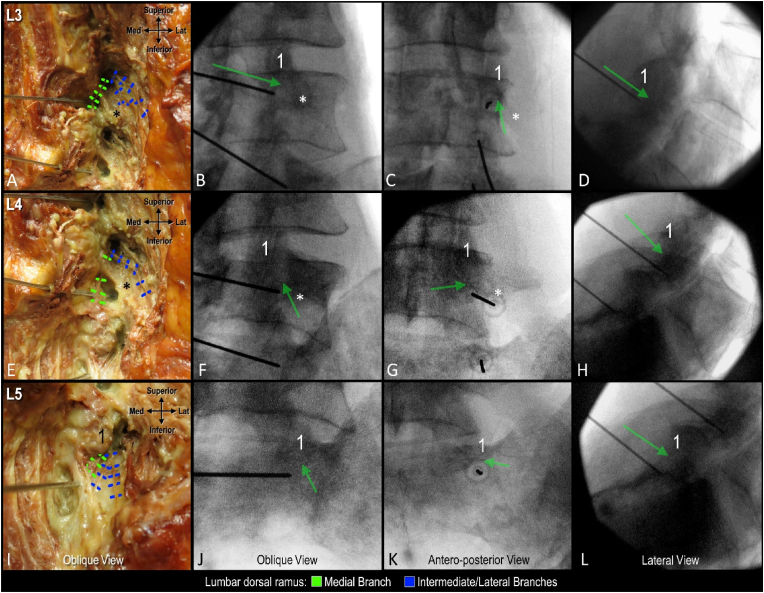
**Anatomy informed optimization of fluoroscopy-guided cannula placement.** A. Dissection showing relationship of cannula placement relative to branches of the lumbar dorsal ramus. B-D. Corresponding fluoroscopic images of cannula placement targeting L3 medial branch. E. Dissection showing relationship of cannula targeting L4 medial branch. F-H. Fluoroscopic images of cannula placement targeting L4 medial branch. I. Dissection of placed cannula targeting L5 medial branch. J-L. Corresponding fluoroscopic image of cannula targeting L5 medial branch. Green arrow indicates optimal cannula tip placement within sub-mammillary fossa; 1, mammillary process; asterisk, accessory process.

Fluoroscopic landmarks to refine cannula placement using the sub-mammillary approach were different for the L3 and L4 MB levels compared to L5. At the L3 and L4 MB levels, the optimized landmark to use in the AP view was the mammillary process with placement of the cannula tip inferior and immediately medial to its lateral boundary (Fig. 6C–G, green arrow). In the oblique view, optimized fluoroscopic landmarks include the mamillo-accessory notch and the inferior boundary of the facet joint line. The cannula tip should be positioned immediately inferior and medial to the mamillo-accessory notch and lateral to the inferior boundary of the facet joint line (Fig. 6B and C, green arrow). In the lateral view, the mammillary process and the pedicle were important landmarks. The cannula tip should be placed inferior to the mammillary process and be located approximately at the midpoint of the cephalo-caudal height of the pedicle (Fig. 6D–H, green arrow). For the L5 MB level, in the AP view, the refined landmark for cannula tip placement was inferior and immediately medial to the lateral boundary of the mammillary process of the sacrum (Fig. 6K, green arrow). In the oblique view, placement immediately lateral to the inferior boundary of the L5/S1 facet joint was identified as a refined fluoroscopic landmark (Fig. 6J). On the lateral view, the inferior boundary of the mammillary process of sacrum was identified as a refined landmark (Fig. 6L).

## 4 Discussion

Robust anatomical evidence is critical to inform image-guided interventions. In the present study, a novel lumbar MB RFA technique targeting the sub-mammillary fossa was investigated in cadaveric models. Utilizing anatomical dissections, fluoroscopic imaging, and high-fidelity 3D modeling, this study provides foundational insights into the feasibility of the sub-mammillary approach for lumbar MB RFA. The correlational findings of the current study provide clinicians with pertinent knowledge to implement the sub-mammillary approach for lumbar MB RFA into their clinical practice. Anatomical research, to validate innovative techniques, is essential in optimizing technical performance and provides a valuable resource to enhance knowledge translation into the clinical setting. Given that the initial technique achieved a complete neural capture rate of below 50 %, further refinements were proposed in this study. These findings suggest that the refined approach to the sub-mammillary fossa may serve as a target in cases where conventional approaches are limited such as severe scoliosis, facet hypertrophy, or spondylolisthesis. Moreover, the sub-mammillary fossa may be used in two lesion lumbar MB RFA techniques to enhance patient outcomes by extending the length or more reliably capture the nerve. Relevant anatomy-fluoroscopy relationships and technical considerations are further discussed below.

### 4.1 Relevant anatomy and fluoroscopic landmarks

**Fig. 7:**
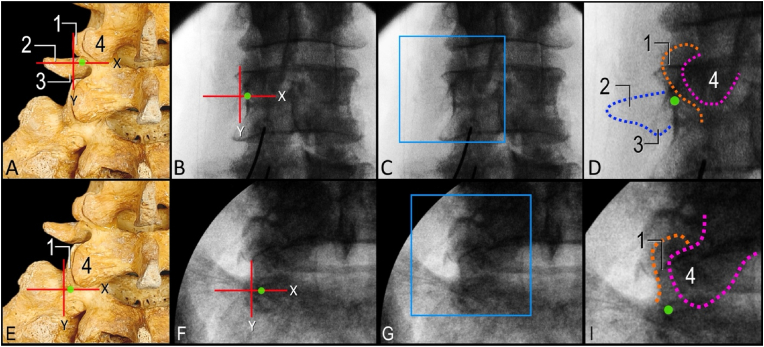
**Proposed fluoroscopic approach to localize optimal sub-mammillary fossa target site in anterior-posterior view.** A. Bony anatomy of lumbosacral spine with targeting overlay for L4 medial branch. B. Fluoroscopic image of L4 vertebra with targeting overlay. C. Fluoroscopic image of L4 vertebra. D. Inset of blue box in panel C showing relevant bony contours. E. Bony anatomy of lumbosacral spine with targeting overlay for L5 medial branch. F. Fluoroscopic image of L5/Sacrum facet joint with targeting overlay. G. Fluoroscopic image of L5/Sacrum facet joint. I. Inset of blue box in panel G showing relevant bony contours. Green circle indicates optimal target site at superior part of sub-mammillary fossa note it's position immediately below mammillary process (1); Blue dashed curve, contour of transverse process (2); Orange dashed curve, contour of superior articular process (SAP) of L4 vertebra and sacrum; Pink dashed curve, contour of inferior articular process (4); 3, accessory process; X, horizontal line parallel with superior vertebral endplate at the level of caudal end of facet joint; Y, vertical line perpendicular with line X at the lateral border of the mammillary process/SAP.

Previous anatomical studies have described the consistent relationship of the MB with the lateral neck of the SAP [[4], [5], [6]]. These studies informed the parallel and perpendicular lumbar MB RFA techniques where the lateral neck of the SAP is the anatomical landmark used to target the nerve [[14], [15], [16]]. In more recent literature, understanding of the relationship of the MB with relevant anatomy has expanded and led to the identification of the sub-mammillary fossa as an additional anatomical landmark of interest [10,12]. Prior to widespread clinical adoption, this novel sub-mammillary fossa landmark should be further assessed to ensure the optimal technique is developed to maximize nerve capture. In the current cannula placement study, the anatomical feasibility and accuracy of targeting the MB using the sub-mammillary landmark were assessed. Following cannula placement, the course of the MB was dissected and traced as far as its termination in the facet joint capsules. Although there was variability in branching patterns, the MB was consistently found to course through the mamillo-accessory notch and the sub-mammillary fossa where it demonstrated variable branching patterns to supply the multifidus and facet joints. Due to the variability in nerve branching, the exact location of placement of the cannula tip within the sub-mammillary fossa had implications on capture rates. Based on the dissected specimens and high-fidelity 3D modelling, the optimal target site for cannula placement was the superior aspect of the sub-mammillary fossa — near the mamillo-accessory notch and inferior border of the mammillary process. Positioning the RFA lesion at this anatomical location is optimal because the MB has not yet divided into branches or has just begun to divide making it more likely to capture the entire nerve and/or all its branches. Therefore, the superior part of the sub-mammillary fossa was identified as the optimal anatomical landmark when targeting the MB using the sub-mammillary approach. Based on the anatomical findings of the current study, we propose a refined fluoroscopically guided approach to target the superior part of the sub-mammillary fossa. In the AP view, two intersecting lines can be used to approximate the optimal target site. First, a horizontal line (parallel to the squared superior vertebral endplate) is drawn at the level of the distal end of the facet joint (Fig. 7, red line labelled X). A second vertical line (perpendicular to X) is placed at the lateral boundary of the mammillary process/SAP (Fig. 7, red line labelled Y). The optimal target site (superior aspect of the sub-mammillary fossa) is located along the X-axis, medial to the point of intersection between lines X and Y (Fig. 7, green circle). Further investigation is needed to assess the anatomical feasibility of the proposed technique for refined cannula placement and to evaluate the ideal appearance on oblique and lateral fluoroscopic views.

### 4.2 Technical considerations

In the current anatomical study, dissection, imaging correlation, and high-fidelity modeling methodology were used to define fluoroscopic landmarks to optimize cannula placement for reliable capture of the MB in the sub-mammillary fossa (Fig. 7). Three important technical considerations related to cannula placement were identified during the current anatomy optimization study. First, due to the orientation of the MB within the sub-mammillary fossa, a perpendicular approach is necessary. Expanded or multi-tined lesions are ideal when using a perpendicular approach to ensure adequate nerve capture. In the present study, an internally-cooled monopolar RF lesion was modeled to assess potential nerve capture rate when targeting the sub-mammillary fossa. Based on published data [13], the near spherical geometry of an internally-cooled monopolar lesion suggests angle independence with regard to orientation relative to the MB. Therefore, the accurate placement of the lesion relative to the nerve target is more important than cannula trajectory. This is consistent with the findings of the current study where placement of the cannula within the sub-mammillary fossa impacted MB capture rate. Second, when the cannula tip is positioned too medial relative to the mammillary process, the frequency of complete nerve capture decreases due to the MB dividing and separating into additional branches within the sub-mammillary fossa. To increase the likelihood of capturing all branches or the MB trunk, the cannula tip placement should be inferior and immediately medial to lateral boundary of mammillary process (as seen on AP fluoroscopic imaging). Third, when the cannula is positioned too inferior relative to the mammillary process, there is a greater risk of completely failing to capture the MB. Due to the geometry of the sub-mammillary fossa, the depth to contact the periosteum gradually increases caudally. Therefore, if cannula placement is too inferior, while in contact with the periosteum, the greater depth may result in the cannula tip and lesion being placed anterior to the MB and missing the nerve target. It is possible that too caudal placement of the cannula within the sub-mammillary fossa would result in the lesion being closer to the intervertebral foramen/nerve root than intended, and thus should generally be avoided. To ensure optimal targeting, the cannula tip should be placed immediately inferior to mammillary process, and for the L3 and L4 MB levels, approximately at the midpoint of the cephalocaudal pedicle height (as seen on lateral fluoroscopic imaging). However, in scenarios where osteophytes are present, the cannula may have to be directed into a more inferior position within the sub-mammillary fossa. A potential technical modification (for safety and effectiveness) would be to retract the cannula tip off the periosteum (posterior to the intervertebral foramen) allowing the lesion to be aligned with the MB at a safe distance from the exiting spinal nerve.

### 4.3 Limitations

The current anatomy optimization study is limited by a small sample size. However, it meets the minimum recommended sample size of 12 for pilot studies with no previous data [17]. Furthermore, the findings of this study provide useful information to develop new research questions and inform future clinical studies to improve patient care. A second limitation is that, as an anatomy study, the findings and technical considerations discussed are postulations that require further clinical investigation. Future studies should include anatomical and clinical studies to further investigate the safety and effectiveness of the refined fluoroscopic landmarks proposed in the current study. The outcome of future studies will determine if further investment of resources in the form of a randomized clinical trial is warranted.

## 5 Conclusions

This anatomy optimization study demonstrates that targeting the sub-mammillary fossa to capture the lumbar MB is technically feasible. Using dissection, imaging correlation, and high-fidelity 3D modeling, we propose an optimized cannula placement technique with associated fluoroscopic landmarks. Refined fluoroscopic landmarks include cannula placement: inferior and medial to the lateral boundary of mammillary process (AP view); inferior and medial to the mamillo-accessory notch/lateral to the inferior boundary of facet joint line (oblique view); immediately inferior to the mammillary process (lateral view). Further research is necessary to confirm the accuracy, safety, and effectiveness of targeting the sub-mammillary fossa using these refined fluoroscopic landmarks.

## Declaration of competing interest

The authors declare the following financial interests/personal relationships which may be considered as potential competing interests:

Eldon Loh reports financial support was provided by Avanos Medical Inc. Zachary McCormick reports a relationship with Avanos Medical Inc that includes: funding grants. Zachary McCormick reports a relationship with 10.13039/100008497Boston Scientific Corporation that includes: funding grants. Zachary McCormick reports a relationship with Saol Therapeutics that includes: funding grants. Zachary McCormick reports a relationship with Spine Biopharma that includes: funding grants. Zachary McCormick reports a relationship with 10.13039/100019735SPR Therapeutics Inc that includes: funding grants. Zachary McCormick reports a relationship with Stratus Medical 10.13039/100023970LLC that includes: funding grants. Zachary McCormick reports a relationship with Stryker that includes: consulting or advisory. Zachary McCormick reports a relationship with Saol Therapeutics that includes: consulting or advisory. Zachary McCormick reports a relationship with International Pain and Spine Intervention Society that includes: board membership. John Tran reports a relationship with International Pain and Spine Intervention Society that includes: funding grants. John Tran reports a relationship with Avanos Medical Inc that includes: funding grants. Aaron Conger reports a relationship with Stratus Medical 10.13039/100023970LLC that includes: funding grants. Aaron Conger reports a relationship with International Pain and Spine Intervention Society that includes: funding grants. Eldon Loh reports a relationship with Avanos Medical Inc that includes: funding grants. Eldon Loh reports a relationship with International Pain and Spine Intervention Society that includes: funding grants. If there are other authors, they declare that they have no known competing financial interests or personal relationships that could have appeared to influence the work reported in this paper.
